# Event-Related Potential Responses to Beloved and Familiar Faces in Different Marriage Styles: Evidence from Mosuo Subjects

**DOI:** 10.3389/fpsyg.2016.00159

**Published:** 2016-02-17

**Authors:** Haiyan Wu, Li Luo, Junqiang Dai, Suyong Yang, Naiyi Wang, Yue-jia Luo

**Affiliations:** ^1^Key Laboratory of Behavioral Science, Institute of Psychology, Chinese Academy of SciencesBeijing, China; ^2^State Key Laboratory of Cognitive Neuroscience and Learning, Beijing Normal UniversityBeijing, China; ^3^School of Education, Guangzhou UniversityGuangzhou, China; ^4^Key Laboratory of Exercise and Health Sciences of Ministry of Education, Shanghai University of SportShanghai, China; ^5^Institute of Educational Psychology and School Counselling, Beijing Normal UniversityBeijing, China; ^6^Institute of Affective and Social Neuroscience, College of Psychology and Sociology, Shenzhen UniversityShenzhen, China; ^7^Institute of NeuroscienceShenzhen, China

**Keywords:** love, affective processing, familiar faces, event-related potentials

## Abstract

Research on familiar face recognition has largely focused on the neural correlates of recognizing a beloved partner or family member. However, no research has explored the effect of marriage style on the recognition of a beloved partner’s face, especially in matriarchal societies. Here, we examined the time course of event-related potentials (ERP) in response to the face of a beloved partner, sibling, or unknown person in a sample of individuals from the matriarchal Mosuo tribe. Two groups were assessed: intermarriage and walking marriage groups (i.e., couples in a committed relationship who do not cohabitate during the daytime). In agreement with previous reports, ERP results revealed more positive VPP, N250, and P300 waveforms for beloved faces than sibling faces in both groups. Moreover, P300 was more positive for beloved partner versus sibling faces; however, this difference emerged at fronto-central sites for the walking marriage group and at posterior sites for the intermarriage group. Overall, we observed that marriage style affects the later stage processing of a beloved partner’s face, and this may be associated with greater affective arousal and familiarity.

## Introduction

The experience of romantic love allows people to build passionate and intimate relationships. Based on previous theory, passion in romantic love is affected by intimacy and relationship duration (Baumeister, 1999; Graham, 2011). Generally, the early stages of romantic love involve intense and passionate emotions, and are later followed by companionate love and the formation of a stable commitment. Romantic love in a relationship is characterized by attachment, care-giving, and sexual attraction (Mikulincer and Goodman, 2006). Several neuroscience studies have proposed that the experience of love involves a neural network distinct from networks that process stimuli relevant to friendship or parental relationships (Bartels and Zeki, 2000, 2004; Aron et al., 2005; Ortigue et al., 2007). Other investigations have confirmed that the affective brain network (Bartels and Zeki, 2004) and reward pathway are correlated with the experience of romantic love (Fisher et al., 2002, 2005; Aron et al., 2005; Zeki and Romaya, 2010; Xu et al., 2011; Acevedo et al., 2012).

Event-related potential (ERP) studies on the visual processing of a beloved partner’s face have provided temporal information regarding the dynamic neural mechanisms underlying familiar face perception. Numerous face-processing studies have identified several early face-related ERP components, including N170, VPP (or P2), and N200 (Bruce and Young, 1986; Luo et al., 2010). P300 has also been identified as sensitive to facial familiarity and emotional arousal, and is an important identifier for self-relevant processing (Grasso et al., 2009; Grasso and Simons, 2011). Previous ERP studies assessing the recognition of a beloved partner’s face have demonstrated that beloved faces elicit a more positive P300 or late positive potential (LPP) than control faces. For example, the LPP was larger when viewing beloved faces than viewing friends’ faces, which was interpreted as a greater reflection of motivational attention toward the beloved face (Langeslag et al., 2007). A subsequent study utilizing an oddball paradigm to dissociate love-related attention from task-related attention also found that P300 was more positive for beloved faces than for friends’ faces, irrespective of whether the beloved face was the target or the distracter stimulus (Langeslag et al., 2008). The authors concluded that perception of a beloved face is accompanied by increased motivational attention for evolutionary reasons (i.e., reproductive). Additionally, it has been proposed that perception of a beloved face is associated with enhanced affective processing (Vico et al., 2006, 2010; Vila et al., 2006). A popular view suggests that emotional stimuli evoke a more positive P300 than neutral stimuli do (Schupp et al., 2004; Pollatos et al., 2005; Eimer and Holmes, 2007). To test this hypothesis, Vico et al. (2010) measured peripheral and central electrophysiological indices, including EEG, heart rate, skin conductance, and zygomatic activity, when subjects viewed five face categories: neutral, unknown, famous, babies, and beloved. Results showed that P300 could differentiate a beloved face from other faces, and furthermore related a larger P300 to the allocation of greater attentional resources. In agreement, it is documented that beloved faces evoke greater arousal of positive emotions (Guerra et al., 2011b). Therefore, despite inconsistent interpretations of P300, there exists a clear association of P300 with the processing of beloved and familiar faces.

The majority of the aforementioned studies placed a focus on passionate or intense romantic love by using the faces of dating partners, where dating can be assumed to describe the early stage of a romantic relationship (de Boer et al., 2012; Langeslag et al., 2015). A recent fMRI study explored the neural correlates of long-term romantic love by evaluating participants that had been married for more than 21 years (Acevedo et al., 2012). Results indicated that activation in reward regions (e.g., the ventral tegmental area and dorsal striatum) is similar in early stage love and long-term love. However, long-term love also involves attachment and pair-bonding-related brain networks (Acevedo et al., 2012). Therefore, it can be hypothesized that brain responses toward beloved faces shift with increasing intimacy and the stage of the relationship. However, few studies have examined brain responses following the recognition of a beloved partner in the context of a long-term relationship, wherein the spouse may be perceived as a family member. In consideration of “love phases” (de Boer et al., 2012), the present study included participants whose relationships had lasted at least seven years (Sternberg, 1987; García, 1998). Therefore, the first aim of the present study was to investigate neural face recognition responses among companionate love partners in stable relationships.

To our knowledge, few studies have investigated whether marriage style modulates the recognition of beloved and familiar faces. China’s Mosuo tribe, which follows a matriarchal culture, provides a unique opportunity for the investigation of marriage style and partner face recognition. In the Mosuo tribe, women are often the head of the household, and inheritance is conveyed through the female line. More interestingly, members of the Muoso tribe are allowed to select between two marriage styles. Several members of the Mosuo tribe maintain a “walking marriage” lifestyle in which there are no husbands or wives, and romantic partners do not live together during the daytime (Yuan and Mitchell, 2000; Walsh, 2001). For instance, a man will stay with his partner during the night and return home early the next morning. The “walking marriage” relationship is somewhat secretive and accordingly does not include economic or childbearing responsibilities. Instead, siblings live together and rear each other’s children, forming a family unit. In contrast, other Mosuo tribe members choose “intermarriage”, which more closely represents a typical modern marriage. In an intermarriage, the married couple cohabitates and forms a core family unit that includes a husband, a wife, and children. Compared to intermarriage, a romantic partner in a walking marriage may be met with higher novelty and arousal. Therefore, different marriage and cohabitation styles may be associated with different neural responses to partner and family member faces. Specifically, we predicted that familiarity responses to a sibling should be higher for people who practice walking marriage than for people who practice intermarriage.

The present study used sibling faces as contrast stimuli to control for familiarity, age, gender, and affective affiliation. To explore neural responses to a beloved partner in Mosuo tribe members, we compared ERP responses to three types of faces (partners, siblings, and unknown persons) during a face discrimination task. We hypothesized that faces of a beloved partner would engender more attention or affective processing than sibling faces. Additionally, we predicted that familiarity processing of a family member’s face would be modulated by marriage style.

## Materials and Methods

### Participants

Forty-five healthy adult Mosuo tribe members participated in the study as paid volunteers. Four participants were excluded due to excessive artifacts or missing data, leaving 41 participants in the following statistical analysis. The walking marriage group consisted of 10 males (*M* = 37.5 years, *SD* = 2.72) and 10 females (*M* = 34.4 years, *SD* = 5.89). The intermarriage group consisted of 12 males (*M* = 36.2 years, *SD* = 3.1) and 9 females (*M* = 33.4 years, *SD* = 6.44). All participants were in a stable relationship for more than 7 years, had children with their partners, and reported an exclusive relationship. Signed informed consent was obtained from all subjects prior to testing in accordance with the Beijing Normal University Review Board guidelines.

### Stimuli

For each participant, the stimulus set included seven digital images (the face of one’s heterosexual lover, the face of one’s opposite-sex sibling, and five faces of unknown opposite-sex Mosuo people). All photographs were taken before the experiment using the same digital camera and background. Faces showed a neutral expression and were processed by Adobe Photoshop CS4 to match brightness, contrast, and size within the stimulus set.

### Procedure

Participants were seated 70 cm from the computer screen and stimuli were presented in the center of a 14-inch screen with a visual angle of 4.3° × 4.6°. A modified oddball and choice reaction paradigm was utilized. The beloved and sibling faces were targets, and the stranger faces were non-target stimuli. Participants were asked to press “F” or “J” in response to their beloved partner or sibling, respectively. The response button was counterbalanced across participants. That is, half of the participants pressed “F” to indicate a beloved partner while the other half pressed “J” to indicate a beloved partner. To evaluate P300, we also manipulated the ratio of face category presentation to be 1:1:5 (beloved partner vs. sibling vs. stranger) for three face categories. Each face was presented 60 times; accordingly, the study consisted of 420 trials. In each trial, a white fixation cross was first presented on a black background for 300 ms, followed by a randomly varied interval of 300–500 ms. Subsequently, a face was presented for 3000 ms and the participants were asked to respond to the target stimulus as soon as possible. The face disappeared upon response within the 3000 ms interval. The trial ended with a randomly varied interval of 300–500 ms.

### Electroencephalogram Recording and Data Analysis

**FIGURE 1:**
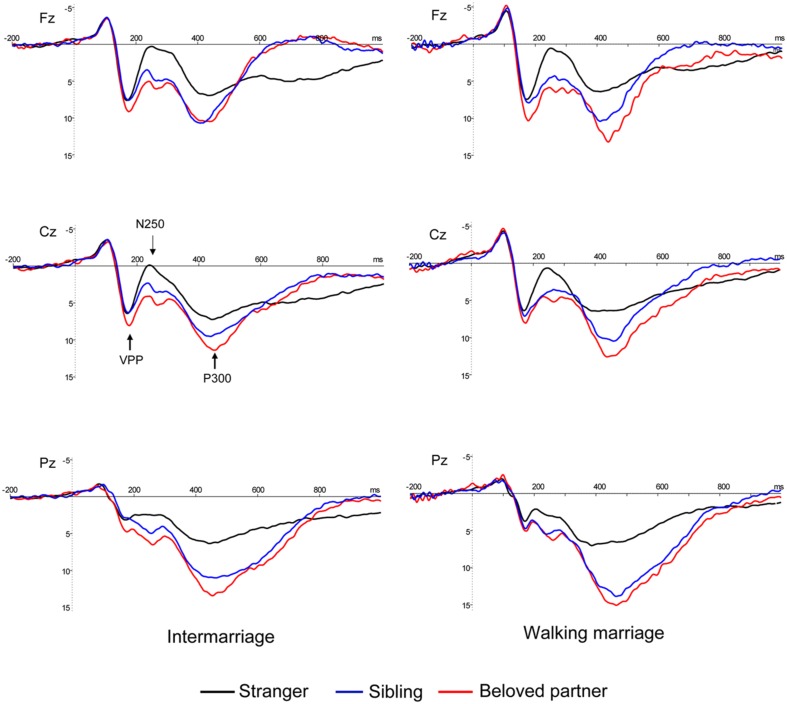
**Grand average ERPs at Fz, Cz, and Pz for two groups.** Familiar faces (beloved and sibling) evoked more positive brain potentials than strangers from 200 to 600 ms for all sites. P300 (marked gray) was more positive for beloved face than sibling for both groups.

Electroencephalograms (EEGs) were recorded using a 64-channel BrainAmp MR with online reference to the left mastoid. Vertical electrooculograms (VEOGs) were recorded from two electrodes positioned above and below the left eye, and horizontal electrooculograms (HEOG) were recorded from two laterally placed electrodes for both eyes. All electrode impedance was maintained below 10 kΩ and the EEG signals were recorded with a band pass of 0.01–100 Hz and sampled at 500 Hz/channel. All electrodes were re-referenced to the average of the bilateral mastoids and filtered oﬄine with a low pass of 30 Hz. EEGs were segmented from 200 ms prior to stimulus presentation until 1000 ms post-stimulus presentation. Trials containing blinks or eye movements (±80 μV) were excluded. The mean left trial number was 53.02 and 54.43 for the beloved partner and sibling conditions, respectively. The grand average ERPs suggested that the face of a beloved partner evoked a more positive potential than the face of a sibling from 200–600 ms (see **Figure 1**). We also observed VPP and N250 over fronto-central sites (Cz, C3, C4, Fz, F3, F4, FCz, FC3, and FC4). Therefore, we first averaged an individual’s mean ERP over fronto-central sites, and then interpreted the local maximum between 150 and 200 ms to be the latency of VPP. Subsequently the amplitude at that specific latency was taken at each individual electrode to calculate amplitude of VPP. We also interpreted the local minimum between 200 and 300 ms to be the latency of N250, and the amplitude at that specific latency was qualified as the amplitude of N250. Considering that the trial number of stranger faces was far greater than that of the other two conditions, and did not require a motor response, we excluded stranger faces from the ERP analysis and focused on the distinctions between beloved partner and sibling face recognition. VPP and N250 were evaluated using a mixed ANOVA with group (walking marriage vs. intermarriage) as the between-subject variable and face category (beloved partner vs. sibling) as the within-subject variable. Given that P300 is a widely distributed component, and that previous studies have dissociated anterior P300 from posterior P300 (Halgren et al., 1998; Friedman et al., 2001; Gaeta et al., 2003; Bobes et al., 2007; Polich, 2007; Cano et al., 2009), we selected Cz, C3, C4, FCz, FC3, FC4, Fz, F3, and F4 in order to analyze the anterior P300 (mean amplitude, 350–600 ms), and Pz, P3, P4, POz, PO3, PO4, CPz, CP3, CP4 in order to analyze the posterior P300 (mean amplitude, 350–600 ms). The P300 amplitude was evaluated using a mixed ANOVA with group (walking marriage vs. intermarriage) as the between-subject variable and face category (beloved partner vs. sibling) and location (anterior vs. posterior) as within-subject variables.

## Results

### Behavioral Results

Incorrect trials and individual response times (RTs) exceeding 3-times the standard deviation (*SD*) were excluded (less than 2%). Furthermore, preliminary analyses revealed no significant effect of gender (all *F*s < 3, *p*s > 0.09) on any variable. Thus, all analyses were collapsed across gender. Accuracy and intra-individual mean RTs within categories were computed and entered into separate mixed two-way ANOVAs (face category by group). Since no significant effects were identified, we conducted planned paired *t*-tests (beloved partner vs. sibling) to examine within-group differences.

Response times were significantly different for beloved versus sibling face discrimination in the walking marriage group (*t*_19_ = 2.66, *p* < 0.05). Walking marriage participants showed longer response times for the beloved face (*M* = 671 ms, *SE* = 23.48) as compared to the sibling face (*M* = 653 ms, *SE* = 21.24). No significant differences were observed in the intermarriage group.

#### VPP

The ANOVA for frontal–central VPP amplitude revealed a significant main effect of face category, *F*(1,39) = 14.73, *p <* 0.001, $\eta_{p}^{2}$ = 0.27, indicating that beloved faces elicited larger VPPs (*M* = 9.67 μV, *SE* = 0.69) than sibling faces (*M* = 8.24 μV, *SE* = 0.69). No significant group effect was observed.

#### N250

Analyses of N250 also showed a significant main effect of face category, *F*(1,39) = 5.71, *p <* 0.05, $\eta_{p}^{2}$ = 0.13. Sibling faces evoked a larger N250 (*M* = 0.26 μV, *SE* = 0.74) than beloved partner faces (*M* = 2.19 μV, *SE* = 0.70).

Considering the observed effect of face category on VPP, we also measured the peak-peak amplitude of N250 to exclude any VPP influences. Results indicated no significant effect of face category and no group effect was observed, *F*s < 0.84, *p*s > 0.37.

#### P300

P300 amplitude was evaluated using a mixed ANOVA with group (walking marriage vs. intermarriage) as a between-subject variable and face category (beloved vs. sibling) and location (anterior vs. posterior) as within-subject variables. We found a significant main effect of face category, *F*(1,39) = 18.98, *p* < 0.01, $\eta_{p}^{2}$ = 0.33, indicating an overall larger P300 for beloved faces. There was also a significant location effect, *F*(1,39) = 10.51, *p* < 0.01, $\eta_{p}^{2}$ = 0.21, which identified a larger P300 at anterior sites. While no significant group effect was observed, we did identify a significant three-way interaction, *F*(1,39) = 5.77, *p* < 0.05, $\eta_{p}^{2}$ = 0.13. *Post hoc* analyses indicated that, for the intermarriage group, the posterior P300 amplitude was larger for beloved faces (*M* = 9.86 μV, *SE* = 1.17) than sibling faces (*M* = 8.78 μV, *SE* = 1.14). For the walking marriage group, the anterior P300 amplitude was larger for beloved faces (*M* = 9.93 μV, *SE* = 1.22) than sibling faces (*M* = 8.75 μV, *SE* = 1.23).

**FIGURE 2:**
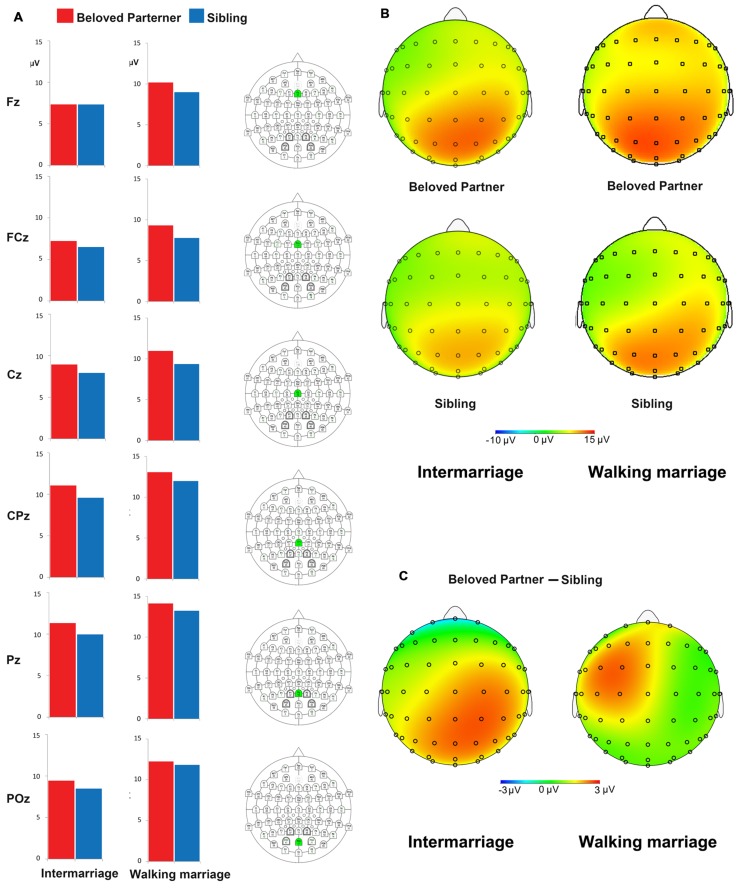
**(A)** The P300 amplitude in the midline electrodes. The bars indicates the mean P300 amplitude at Fz, FCz, Cz, CPz, Pz, and POz (corresponding electrode was marked as green in column III); **(B)** The topography of P300 for grand averaged face conditions (beloved partner vs. sibling) for two groups; **(C)** The topography of difference wave of P300 (beloved partner vs. sibling) for two groups. The topography map indicated that the beloved partner vs. sibling P300 difference mainly occurs at posterior sites (also at some frontal sites) for intermarriage group while the difference only occurs at anterior sites for walking marriage group. The figure indicates that the beloved partner vs. sibling difference changes from the anterior to posterior sites. Such difference is larger at posterior sites for intermarriage group, while the difference is larger at anterior sites for walking marriage group.

Electrophysiological results for the P300 component in both groups and scalp voltage topography for the beloved *vs.* sibling difference waves (P300) are summarized in **Figure 2**. The topography map confirmed that the beloved vs. sibling P300 difference occurred mainly at posterior sites in the intermarriage group, while P300 differences only occurred at anterior sites in the walking marriage group. Interestingly, we primarily identified a differential pattern between beloved and sibling faces over the left frontal brain area. To validate this observation, we conducted an electrode side (left: F3, FC3, and C3 vs. right: F4, FC4, and C4) × face type ANOVA on the anterior P300. Results showed a significant electrode side effect on the anterior P300, *F*(1,39) = 5.77, *p* < 0.05, $\eta_{p}^{2}$ = 0.13, such that a more positive P300 was observed in the right fronto-central area versus the left.

## Discussion

The present study examined ERP responses to the face of a beloved partner or sibling in Mosuo tribe members practicing walking marriage or intermarriage. Results indicated that individuals in the walking marriage group exhibited slower response times to beloved faces than sibling faces. One possible interpretation is that walking marriages represent a less secure relationship than a familial sibling relationship. The potential secrecy associated with a walking marriage may cause individuals to associate a beloved face with greater novelty, and thus lead to slower reaction times during an explicit face judgment task. Notably, we observed no differences in recognition accuracy between groups or between faces. This may suggest a “ceiling effect” in the face task, as mean accuracy (96.9%) was very high across groups and conditions.

With regard to ERP results, beloved faces were associated with more positive ERP potentials than sibling faces from 200 ms to the late processing stage (VPP, N250, and P300). VPP at centro-frontal sites, occurring between 150 and 200 ms post-stimulus presentation, is often regarded as similar to N170 (Rossion et al., 1999, 2003; Itier and Taylor, 2002; Jemel et al., 2003; Joyce and Rossion, 2005). Although some studies have reported that VPP is not modulated by emotional stimuli (Rossignol et al., 2005) or familiarity (Rossignol et al., 2005; Sui et al., 2006; Marzi and Viggiano, 2007), results from expression processing studies suggest that VPP is subject to modulation by affectively salient stimuli (Ashley et al., 2004; Williams et al., 2006; Foti et al., 2010). Therefore, enhanced VPP amplitudes observed in response to a beloved partner’s face might reflect affective salience in early perception.

VPP enhancement in response to a beloved face likely influenced the effect observed for N250, as the peak-peak N250 amplitude revealed no significant effect. N250 (or N300 in some studies) has been reported to be sensitive to facial familiarity (Schweinberger et al., 1995; Tanaka et al., 2006; Krigolson et al., 2009) and affective features (Eimer and Holmes, 2002, 2007). While N250 results are in accordance with previous studies demonstrating that beloved faces (fathers and romantic partners) are associated with smaller N200s (Guerra et al., 2011a), we conjecture that the N250 effect in our study was due to VPP enhancements after viewing the face of a beloved partner.

Clearly, the P300 component discriminated the perception of familiar faces from unknown faces in our study, as familiar faces elicited a more positive P300. This effect is consistent with several previous studies (Langeslag et al., 2007, 2008; Guillaume et al., 2009). Furthermore, enhancements in the amplitude of P300 were greater in response to the face of a beloved partner versus a sibling. We speculate that a beloved partner provides more intimate/self-relevant significance, regardless of marriage type. We did however identify a dissociation of anterior P300 from posterior P300 based on marriage type. An enhanced posterior P300 amplitude was observed for beloved partner versus sibling faces in the intermarriage group, while the anterior P300 amplitude was more positive for beloved partner versus sibling faces in the walking marriage group. The anterior and posterior P300 components may reflect differences in psychological significance. The posterior P300, which is more akin to classic P300 (e.g., peaking at parietal sites), may reflect face familiarity or a memory process that discriminates the familiar from the unfamiliar (Miyakoshi et al., 2007; Guillaume et al., 2009). On this premise, it is unsurprising that the intermarriage group showed widely distributed differences in P300 response to beloved partners versus siblings, especially in the posterior P300 (see **Figure 2**). Compared to the walking marriage group, a beloved partner in the intermarriage group may be of higher familiarity than siblings as a result of cohabitation and child rearing. There was no significant beloved partner versus sibling difference in the posterior P300 for the walking marriage group. This is likely because familiarity was more closely matched between one’s beloved partner and sibling in the walking marriage group, relative to intermarriage group. Consequently, the posterior P300 result could be interpreted as a memory-related component in which a person of higher familiarity is correlated with larger P300 amplitudes.

The anterior P300 over fronto-central sites is more akin to a P3a component, which reflects orientation responses toward novel stimuli (Friedman et al., 2001; Polich, 2007; Weisman et al., 2012). Our most remarkable finding was that the anterior P300 was enhanced in response to beloved partner versus sibling faces in the walking marriage group. This anterior P300 effect fits well with our hypothesis of a partner-viewing novelty effect in the walking marriage group, wherein limited contact (i.e., only at night) produces enhanced arousal toward the beloved partner’s face. The beloved partner versus sibling difference was not significant for the anterior P300 in the intermarriage group. This may be attributed to the cohabitation environment, as intermarried couples have extensive contact with their partner, which is likely to decrease partner-face novelty.

On the other hand, neuroimaging studies have demonstrated that romantic love involves affective state-related brain regions, including the anterior cingulate cortex, the orbitofrontal cortex (OFC), and the striatum/reward system (Aron et al., 2005; Fisher et al., 2005; Xu et al., 2011; Acevedo et al., 2012). These regions, mostly within fronto-central areas, may contribute to the perception of beloved faces. Given the observation of differences in the response of anterior P300 to beloved partner versus sibling faces, we agree with Guerra et al. (2011b) that the frontal P300 may relate more specifically to the perception of a beloved partner’s face.

Interestingly, we also observed overall left-right anterior P300 differences in the walking marriage group for responses to beloved versus sibling faces (see **Figure 2**). Lateralization over left fronto-central cites may be indicative of emotion-motivation system involvement, as asymmetric frontal cortical activity is correlated with affective experience (Harmon-Jones et al., 2011). Specifically, approach motivation is more significantly associated with left frontal cortical activity (Hewig et al., 2004). Another previous study demonstrated that appetitive pictures evoke a larger LPP than neutral pictures over left frontal sites (Gable and Harmon-Jones, 2010). Therefore, our observation that the face of a beloved partner produces a larger P300 over left fronto-central sites is supportive of our interpretation that the anterior P300 reflects an orienting response toward motivational stimuli.

One limitation of present study is the absence of familiarity and affect ratings from both groups. We chose not to collect this data due of the conservative nature of our population and the “taboo” nature of sex-related questions. Therefore, we cannot definitively attribute P300 effects to familiarity or affective feelings. Further studies using subjective ratings and behavioral data are needed to validate our findings. Further, the lack of a normal, non-Mosuo marriage group to control for minority or tribe-related effects limits our ability to make inferences regarding P300 in other populations.

In summary, the present study provides a unique examination of companionate and sibling love, and reveals that ERPs can differentiate beloved partners from siblings in the early and late stages of processing. The VPP and N250 ERP components are thought to discriminate the perception of familiar individuals from unfamiliar individuals. In our study, more positive VPP and N250 values were not only observed for familiar versus unknown faces, but also for beloved partner versus sibling faces; thus, these components indicate early face configuration and identification processing of a beloved partner. In line with previous studies, P300 was also sensitive to beloved faces, and furthermore we differentiated anterior and posterior P300s in individuals with different marriage styles. The present study therefore demonstrates that early processing of a beloved partner’s face is conserved across various relationship types, but affective or familiarity processing is subject to divergence in the late phase.

## Author Contributions

YJL and HW conceived the experiment, HW, LL, JD, and SY conducted the experiment, LL andHWanalyzed the results. HW, NW, SY, and YJL wrote the paper. All authors reviewed the manuscript.

## Conflict of Interest Statement

The authors declare that the research was conducted in the absence of any commercial or financial relationships that could be construed as a potential conflict of interest. The reviewer, LH, and handling Editor declared their shared affiliation, and the handling Editor states that the process nevertheless met the standards of a fair and objective review.
